# Abstract of Meteorological Observations for June, 1856, Made at Philadelphia, Pa.

**Published:** 1856-08

**Authors:** James A. Kirkpatrick


*Abstract of Meteorological Observations for June, 1856, made at Philadelphia, Pa. Latitude 39° 57′ 28″ N., Longitude 75° 10′ 40″ W. from Greenwich.* By Prof. James A. Kirkpatrick.

| 1856. June. | Barometer. Daily Mean | Barometer. Mean Daily Range. | Thermom. Daily Mean | Thermom. Mean Daily Range | Rel. Humid. 2 P.M. | Force of Vapor 2 P.M. | Dew Point 2 P.M. | Rain | Prevailing Winds. | Remarks. |
|---|---|---|---|---|---|---|---|---|---|---|
| | Inches. | Inches. | Deg. | Deg. | Hunds. | Inches. | Deg. | Inches. | Points. | |
| 1 | 29.990 | .034 | 66.3 | 11.3 | 49 | .370 | 50.7 | | SW. | Morning cloudy; afternoon and evening clear. *Barometer highest* 30.054. *Thermometer lowest* 54°. |
| 2 | 29.915 | .075 | 76.0 | 9.7 | 45 | .495 | 58.7 | | SW. | Morning and afternoon cloudy; evening clear. |
| 3 | 29.803 | .109 | 82.0 | 6.0 | 46 | .608 | 64.6 | | SW. | Cloudy. |
| 4 | 29.729 | .074 | 84.0 | 2.0 | 48 | .697 | 68.5 | | SW. | Clear. |
| 5 | 29.864 | .146 | 72.3 | 13.0 | 55 | .483 | 58.0 | 0.022 | NE. | Morning clear; noon, strong wind from N. E. continuing about two hours; night, light *rain*. |
| 6 | 29.959 | .098 | 58.3 | 14.0 | 100 | .465 | 57.0 | 1.111 | NE. | Cloudy; *rain* from 10, A.M. to 6, P.M. *Therm. lowest* 54°. |
| 7 | 29.863 | .097 | 68.0 | 9.7 | 66 | .502 | 59.2 | | NE. | Cloudy. |
| 8 | 29.684 | .178 | 70.0 | 2.0 | 76 | .593 | 63.8 | 0.003 | SSW. | Cloudy; 10 to 11, A.M., *rain*, light showers during the afternoon. |
| 9 | 29.627 | .068 | 75.0 | 5.0 | 43 | .429 | 54.7 | | (Var.) | Cloudy. |
| 10 | 29.713 | .086 | 75.3 | 1.7 | 55 | .560 | 62.2 | | SW. | Cloudy. |
| 11 | 29.743 | .036 | 77.7 | 2.3 | 52 | .571 | 62.8 | | SW. | Clear. |
| 12 | 29.758 | .051 | 74.5 | 3.2 | 69 | .638 | 66.0 | | SE. | Morning fog; afternoon and evening cloudy. |
| 13 | 29.677 | .081 | 76.3 | 1.8 | 51 | .520 | 60.1 | | (Var.) | Morning and aft. cloudy; evening clear. |
| 14 | 29.642 | .037 | 75.5 | 0.8 | 62 | .611 | 64.7 | 0.006 | SW. | Cloudy; light *showers* at 6¼, 10 and 11, A.M. |
| 15 | 29.714 | .072 | 72.2 | 3.3 | 43 | .372 | 50.8 | | W. | Morning and evening clear; afternoon cloudy. |
| 16 | 29.876 | .163 | 71.7 | 2.2 | 44 | .381 | 51.5 | | W. | Clear. |
| 17 | 29.907 | .040 | 75.0 | 3.3 | 47 | .464 | 56.9 | | SW. | Cloudy; 10, P.M. *rain* began. |
| 18 | 29.729 | .178 | 74.3 | 2.0 | 72 | .627 | 65.4 | 0.500 | (Var.) | Cloudy; *rain* continued till 12, M. |
| 19 | 29.732 | .041 | 75.0 | 2.7 | 58 | .573 | 62.9 | | (Var.) | Morning and afternoon cloudy; evening clear. |
| 20 | 29.833 | .101 | 79.8 | 4.8 | 50 | .608 | 64.6 | | SW. | Clear. |
| 21 | 29.905 | .072 | 86.2 | 6.3 | 41 | .652 | 66.6 | | W. | Clear. |
| 22 | 29.859 | .049 | 89.0 | 3.2 | 39 | .692 | 68.3 | 0.005 | W. | Morning and evening clear; light *showers* with high wind from 5 to 7, P.M. |
| 23 | 29.882 | .076 | 80.8 | 8.2 | 66 | .703 | 68.8 | 0.016 | (Var.) | Cloudy; light showers at intervals from 8½, A.M. to 3, P.M. |
| 24 | 29.872 | .047 | 77.7 | 3 8 | 49 | ·532 | 60.7 | | (Var.) | Morning and afternoon cloudy; evening clear. |
| 25 | 29.801 | .071 | 76.3 | 2.0 | 70 | .689 | 68.2 | 0.014 | (Var.) | Cloudy; *rain* from 9 to 11, A.M. |
| 26 | 29.718 | .083 | 81.5 | 5.2 | 61 | .761 | 71.1 | | S. | Cloudy. |
| 27 | 29.736 | .065 | 83.0 | 1.8 | 26 | .342 | 48.6 | | NW. | Morning cloudy; afternoon and evening clear. |
| 28 | 29.722 | .086 | 83.7 | 2.0 | 53 | .720 | 69.5 | | SW. | Clear. |
| 29 | 29.585 | .137 | 89.0 | 5.3 | 50 | .818 | 73.3 | | SW. | Clear. *Bar. lowest* 29.553. *Thermometer highest* 98°. |
| 30 | 29.599 | .038 | 90.5 | 1.5 | 50 | .818 | 73.3 | | SW. | Morning cloudy; afternoon and evening clear. |
| Means for June, 1856 | 29.781 | .083 | 77.2 | 4.7 | 55 | .576 | 62.4 | 1.677 | S. 45° 48′ W. 5-100. | |
| Means for June, 5 yrs. | 29.836 | .096 | 73.2 | 4.4 | 55 | .561 | 61.4 | 3.735 | S. 65° 49′ W. 27-1003. | |

